# Profiling the Behavior of Distinct Populations of Head and Neck Cancer Stem Cells

**DOI:** 10.3390/cancers8010007

**Published:** 2016-01-04

**Authors:** Luciana O. Almeida, Douglas M. Guimarães, Cristiane H. Squarize, Rogerio M. Castilho

**Affiliations:** 1 Laboratory of Epithelial Biology, University of Michigan School of Dentistry, Ann Arbor, MI 48109, USA; lubio2001@gmail.com (L.O.A.); douglas_guima@hotmail.com (D.M.G.); csquariz@umich.edu (C.H.S.); 2 Department of Periodontics and Oral Medicine, University of Michigan School of Dentistry, Ann Arbor, MI 48109, USA; 3 Department of Oral Pathology, School of Dentistry, University of Sao Paulo, Sao Paulo, SP 05508-000, Brazil; 4 Comprehensive Cancer Center, University of Michigan, Ann Arbor, MI 48109, USA

**Keywords:** HNSCC, CSC, cancer initiating cells, spheres, holoclones, meroclones, paraclones, ALDH, BMI-1, histone

## Abstract

Cancer stem cells (CSCs) are a subpopulation of tumor cells endowed with self-renewal properties and the capacity to dynamically adapt to physiological changes that occur in the tumor microenvironment. CSCs play a central role in resistance to therapy and long-term disease recurrence. Better characterization and understanding of the available *in vitro* tools to study the biology of CSCs will improve our knowledge of the processes underlying tumor response to therapy, and will help in the screening and development of novel strategies targeting CSCs. We investigated the behavior of different populations of head and neck CSCs grown under ultra-low adhesion conditions. We found that invasion and adhesion differ among tumorsphere subtypes (holospheres, merospheres and paraspheres), and their tumor cell progeny also harbor distinct self-renewal and clonogenic potentials. Furthermore, holospheres contained higher numbers of head and neck CSCs, as detected by the CD44 cancer stem cell marker and aldehyde dehydrogenase (ALDH) enzymatic activity. In addition, holospheres showed reduced proliferation (Ki67), hypoacetylation of histones, and increased expression of the BMI-1 epithelial stem cell marker, suggesting activation of stem cell programs. Collectively, our results suggest that holospheres enrich a specific population of CSCs with enhanced “stemness” and invasive potential.

## 1. Introduction

Head and neck squamous cell carcinoma (HNSCC) is a malignant solid tumor responsible for the deaths of more than 300,000 patients/year worldwide [1]. The economic impact of this disease is considerable, and it is projected that treatment in the United States will cost more than four billion dollars in 2016 [2]. Despite investments in research, prevention, and treatment, the overall survival rate has only marginally improved in the last few decades [3].

Differences in tumor behavior and the acquisition of resistance to available therapy suggest that HNSCC is a heterogeneous disease that dynamically adapts to changes in the tumor microenvironment. Such tumor plasticity arises from different mechanisms, including the acquisition of new mutations, changes in gene expression mediated by epigenetic mechanisms, and the presence of a subpopulation of tumor cells, termed cancer stem cells (CSCs).

CSCs are a small subpopulation of neoplastic cells within the tumor mass that retain high tumorigenic potential and increased self-renewal properties. Similar to normal stem cells, the cell cycle in CSCs is slow, which confers resistance to therapies that primarily target rapidly dividing cells. CSCs are also associated with metastasis, long-term relapse, and the reactivation of tumor malignancy after long periods of inactivity or dormancy. CSCs have been found in many tumors, including those of the breast, colon, prostate, pancreas, brain, and head and neck [4,5,6,7,8,9]. Similar to stem cells, CSCs enrich their numbers by forming colonies advent from single tumor cells. Tumor colonies growing in ultra-low adhesion conditions produce clonal sphere structures, or tumorspheres, that can be subclassified into three morphological categories known as holospheres, merospheres, and paraspheres [10]. Differences in the biology, clonogenic potential, and aggressiveness among the distinct populations of HNSCC spheres likely play a critical role in tumor behavior and resistance to therapy.

Using a series of clonogenic experiments and markers for CSCs, including aldehyde dehydrogenase (ALDH) enzymatic activity and CD44 [11,12,13] and BMI-1 expression, we observed that holospheres are the most common HNSCC tumorsphere subtype. Further, holoclone-driven tumor cells are significantly more efficient in attaching to substrates, invading fibronectin extract, and reconstituting the heterogeneity of CSCs by forming new holospheres, merospheres, and paraspheres. Our work demonstrates that tumor heterogeneity observed in HNSCC is also found among CSCs, and that holoclone-driven CSCs are likely to play an important role in tumor progression, invasion, and resistance to chemotherapy.

## 2. Results

### 2.1. HNSCC Cell Lines Contain Distinct Subpopulations of CSCs That Can Be Enriched by Ultra-Low Attachment Culture Techniques

**Figure 1:**
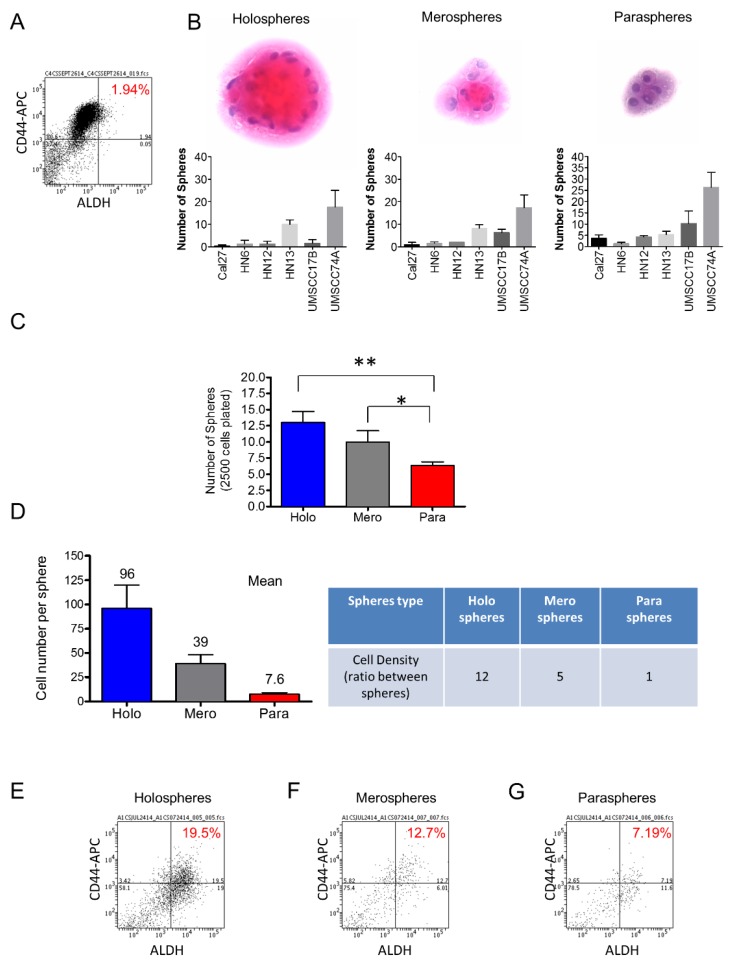
*Ultra-low attachment culture conditions enhance the number of CSCs*. (**A**) Cell sorting to determine the total number of tumor cells from an HNSCC cell line cultured in monolayer. *N*,*N*-diethylaminobenzaldehyde (DEAB) was used to inhibit ALDH activity resulting in the identification of negative cells for ALDH; (**B**) H and E staining of tumor spheres depicting the typical morphological appearance of holospheres, merospheres, and paraspheres. HNSCC tumor cell lines Cal7, HN6, HN12, HN13, UMSCC17B, and UMSCC74A are able to generate holospheres, merospheres, and paraspheres; (**C**) Total number of tumor spheres from a culture of 2.5 × 10^3^ tumor cells under ultra-low adhesion conditions (** *p* < 0.01, * *p* < 0.05); (**D**) Mean of the total number of cells per sphere subtype and the ratio of cellular density between different spheres types; (**E**–**G**) Percentage of tumor cells positive for CD44 and ALDH stem cell markers.

Two of the most promising markers of head and neck CSCs include the enzymatic activity of ALDH and the expression of the CD44 cell surface marker [9,14,15,16]. ALDH levels alone have been associated with high metastatic potential and negatively influence the clinical outcome of solid tumors [17]. We found that HNSCC cells cultured in monolayer had a relatively low percentage of ALDH positive cells (~2%) (Figure 1A). Landmark studies have demonstrated a correlation between stem cell properties and the morphology of colonies generated by single cells from hair follicles [18], epidermal keratinocytes [19], and HNSCC [20], among other tissues. We found that tumor cells derived from six different HNSCC cell lines growing in ultra-low adhesion conditions are able to generate three morphologically distinct sphere populations classified as holospheres, merospheres, and paraspheres (Figure 1B). Interestingly, HNSCC generated a significantly higher number of holospheres (** *p* < 0.01) and merospheres (* *p* < 0.05) compared to paraspheres (Figure 1C). Upon dissociation of individual spheres to single cell suspension, we found that for each tumor cell present in paraspheres (mean 7.6 cells), there were five tumor cells in merospheres (mean 39.3 cells) and 12 tumor cells in holospheres (mean 96 cells), suggesting an increased clonogenic potential of tumor cells to form holospheres and, to some degree, merospheres (Figure 1D). To better understand the differences between sphere subtypes, we examined their CSC content. We separated spheres into holospheres, merospheres and paraspheres by carefully pipetting each sphere subtype from its ultra-low adhesion culture flask and dissociating using trypsin. We then identified head and neck CSCs using CD44 expression and ALDH activity by flow cytometry. Holospheres enriched the population of CD44/ALDH-positive cells ten-fold when compared to the same cell line grown in normal culture conditions (adherent cells) (Figure 1E). Similarly, merospheres enriched the population of CSCs by six-fold (Figure 1F), while paraspheres had their CD44/ALDH-positive cellular population enriched by three-fold (Figure 1G). Interestingly, changes in the CD44/ALDH ratio of tumor spheres compared to tumor cells growing under adherent conditions were observed (Figure 1A,E,F,G). Although unexpected, the increased ratio between ALDH positive cells and CD44 positive cells observed in tumorspheres alludes to the observed enhanced expression of ALDH upon ultra-low adhesion culture conditions. Although we observed great variation in the efficiency of holospheres, merospheres, and paraspheres to accumulate CSCs, all sphere subtypes fostered the expansion of CSCs beyond basal levels. However, the biological implications of this cellular expansion in tumor behavior remain unknown.

### 2.2. Holospheres Progeny Have Increased Adhesion and Spreading Characteristics

**Figure 2:**
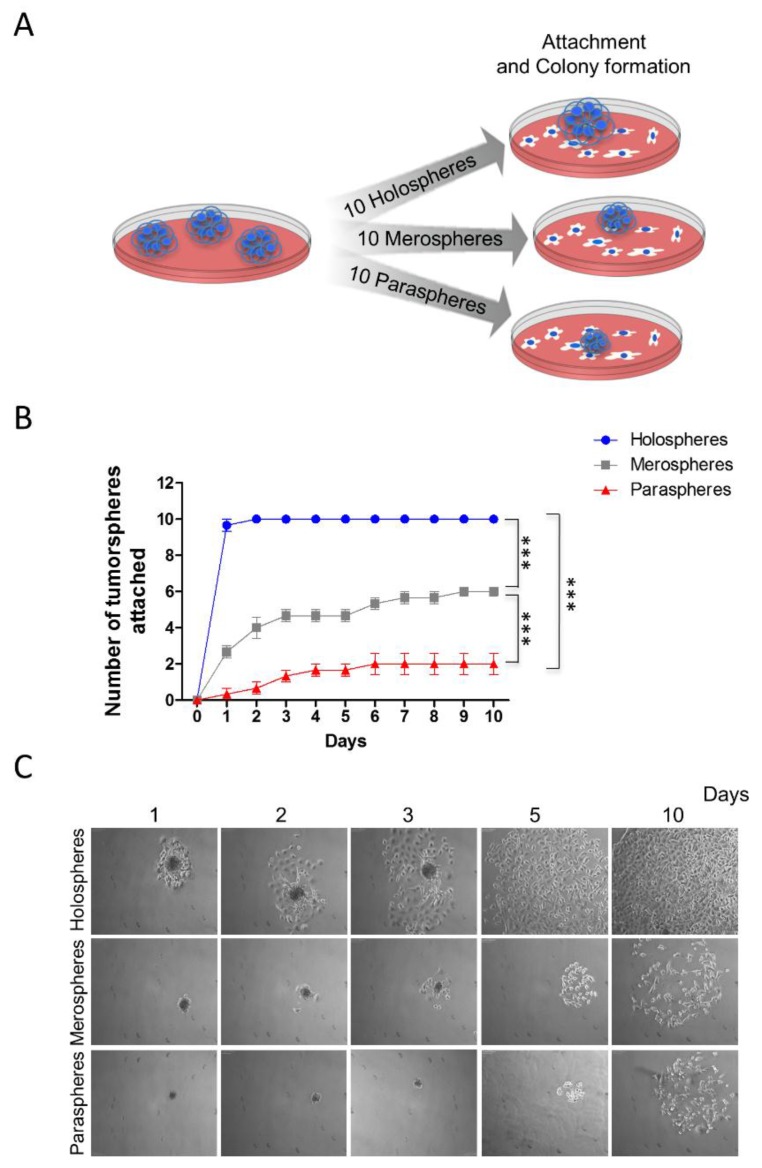
*Increased reattachment and spreading of tumor cells from holospheres*. (**A**) Schematic representation of tumor spheres (*n* = 10) isolated based on morphology (holospheres, merospheres, or paraspheres) and seeded into culture dishes (adherent culture conditions); (**B**) Time course and quantification of adhesion efficiency of tumor spheres (*** *p* < 0.001); (**C**) Representative examples of holospheres, merospheres, and paraspheres adhering and spreading in culture dishes.

CSCs play a major role in tumor resistance to therapy [21,22,23,24]. In addition to chemoresistance and radioresistance, CSCs are also involved in tumor invasion and metastasis [25,26]. We examined the ability of tumorspheres to reattach to substrates after prolonged growth in ultra-low adhesion conditions. Ten clones from holospheres, merospheres and paraspheres were isolated by pipetting and being seeded onto normal culture dishes that allowed cell attachment (Figure 2A). Spheres were followed for 10 days, and the number that attached was quantified. Interestingly, tumorspheres containing increased numbers of CSCs (holospheres) were significantly more efficient at adhering to substrate than spheres containing smaller numbers of CSCs (merospheres and paraspheres) (Figure 2B-blue) (*** *p* < 0.001). All holospheres adhered to substrate within the first two days of culture, and all cells spread out of spheroid bodies by day five (Figure 2C). Merospheres were more efficient (six viable spheres out of 10) than paraspheres at adhering to the new culture substrate (Figure 2B-gray) (*** *p* < 0.001). Paraspheres had the lowest number of spheres successfully attach (*n* = 2) (Figure 2B-red). Initial cellular spread out of the paraclone spheroid body was only observed by day five (Figure 2C).

### 2.3. Tumor Cells Derived from Holospheres and Merospheres Retain the Ability to Generate All Three Subtypes of Spheroid Bodies

**Figure 3:**
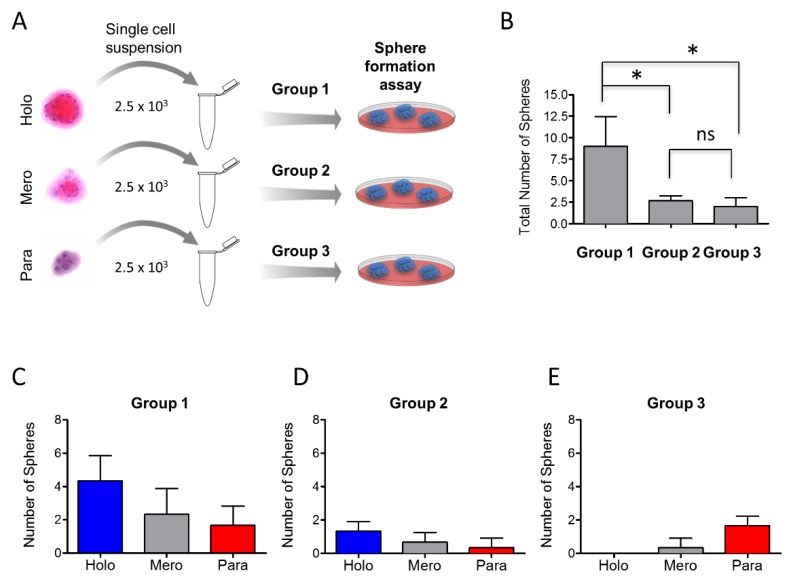
*Holosphere- and merosphere-derived tumor cells generate all 3 types of tumor spheres.* (**A**) Schematic representation of tumor sphere-derived single cells isolated from holospheres (group 1), merospheres (group 2), or paraspheres (group 3) and seeded into ultra-low adhesion culture dishes; (**B**) Quantification of clonogenic potential of each group of tumor cells (* *p* < 0.05). Note that holosphere-derived tumor cells retain higher clonogenic potential compared to merosphere- and parasphere-derived tumor cells; (**C**–**E**) Quantification of tumor spheres derived by group. Note that only holosphere- and merosphere-derived tumor cells regenerate all three types of tumor spheres. Tumor cells from paraspheres do not generate holospheres.

We next examined whether tumor cells derived from holospheres, merospheres, and paraspheres retained similar clonogenic potential to form all three sphere subtypes. Tumorspheres were isolated accordingly by morphology, dissociated into single cell suspensions, and divided into group 1 (holosphere-derived tumor cells), group 2 (merosphere-derived tumor cells), and group 3 (parasphere-derived tumor cells) (Figure 3A). Each group had the same initial cellular density (2.5 × 10^3^ cells). All cells were seeded in ultra-low adhesion plates and grown for five days. Tumor cells in group 1 (holospheres) showed a three-fold increase in the total number of spheres compared to groups 2 and 3 (Figure 3B) (* *p* < 0.05). There was not a significant difference in the number of spheres between groups 2 and 3 (ns *p* > 0.05). We then quantified the number of tumorspheres in each group by morphological appearance. This analysis determines whether tumor cells isolated from different spheroid bodies retain similar clonogenic potential. We found that single cell suspensions from group 1 (holospheres) and group 2 (meropheres) generated all three types of tumorspheres (Figure 3C,D). In contrast, tumor cells from group 3 failed to generate holospheres (Figure 3E). CSCs are comprised of a heterogeneous cellular population with distinct clonogenic potential and likely distinct biological behavior, similar to HNSCC cells.

### 2.4. Holosphere Progeny Retain Long-Term Clonogenic Potential

**Figure 4:**
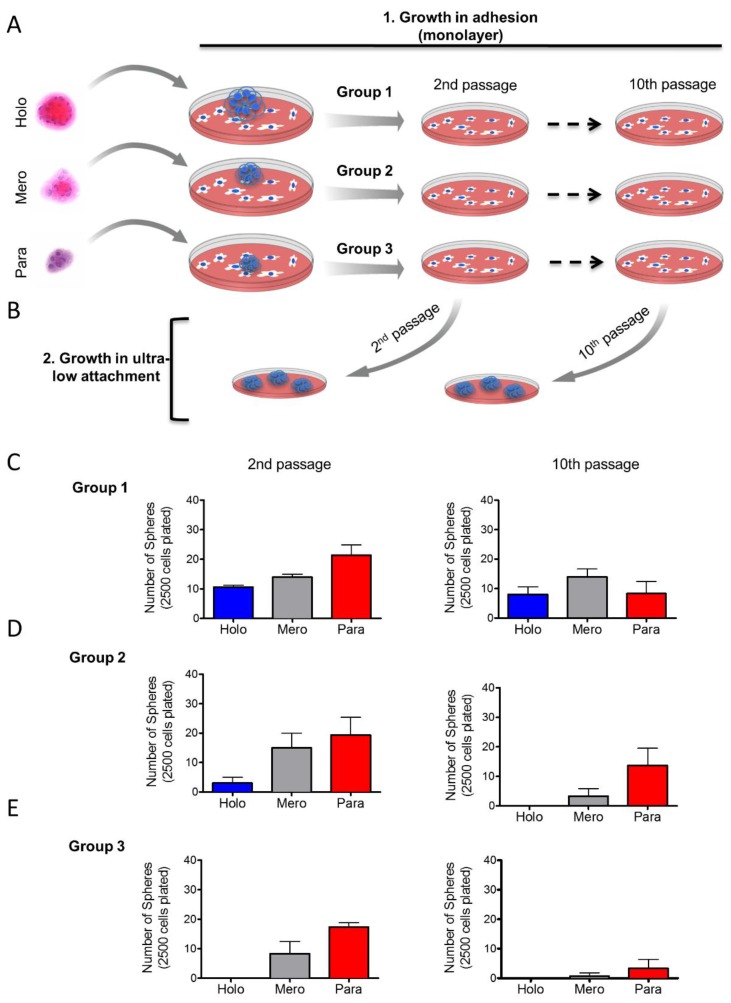
*Long-term maintenance of CSC clonogenic potential.* (**A**) Schematic representation of long-term culture of sphere-derived tumor cells under adhesion conditions. Tumor spheres from holospheres, merospheres, or paraspheres were isolated and seeded into culture in monolayer. Tumor cells from each group were maintained in monolayer culture for up to 10 passages; (**B**) Schematic representation of tumor cells derived from groups 1, 2, and 3 isolated from passages 2 and 10 and seeded into ultra-low adhesion culture dishes; (**C**) Tumor cells from group 1 (initially derived from holospheres) regenerate holospheres, merospheres, and paraspheres by passages 2 and 10; (**D**) Tumor cells from group 2 (initially derived from merospheres) only generated holospheres by passage 2 but failed to generate holospheres by passage 10; (**E**) Tumor cells from group 3 (initially derived from paraspheres) do not regenerate holospheres.

Based on our previous findings, we examined whether tumor cells derived from holospheres, merospheres, or paraspheres retained their clonogenic potential after long-term cell culture in a monolayer. Tumorspheres from holospheres (Group 1, *n* = 10), merospheres (Group 2, *n* = 10) and paraspheres (Group 3, *n* = 10) were isolated and seeded into normal culture dishes in order to adhere. Cells that adhered received fresh media until 70% confluent, at which point they were trypsinized and seeded into new culture dishes. All tumor cells were subcultured in monolayer for up to 10 passages (Figure 4A). At passages 2 and 10, a portion of tumor cells derived from groups 1–3 were seeded into ultra-low adhesion culture dishes to generate tumorspheres (Figure 4B). Holospheres-derived tumor cells (Group 1) retained their clonogenic potential, as demonstrated by their ability to regenerate all three subtypes of spheres following both passages (Figure 4C—Group 1). Like holospheres, merosphere-derived tumor cells (Group 2) retained the ability to reconstitute all three subtypes of spheres after the second passage. However, prolonged subculture of merosphere-derived tumor cells (tenth passage) resulted in their inability to generate holospheres; in addition, they only generated a modest number of merospheres and paraspheres (Figure 4D—Group 2). Parasphere-derived tumor cells (Group 3) had the lowest clonogenic potential. After two passages, paraspheres-derived tumor cells were unable to reconstitute holospheres, and they generated minimal merospheres and paraspheres by passage 10 (Figure 4E). These findings suggest that we isolated CSCs with differing clonogenic potentials through the use of sphere forming assays.

### 2.5. Holospheres Contain Highly Invasive Tumor Cells

**Figure 5:**
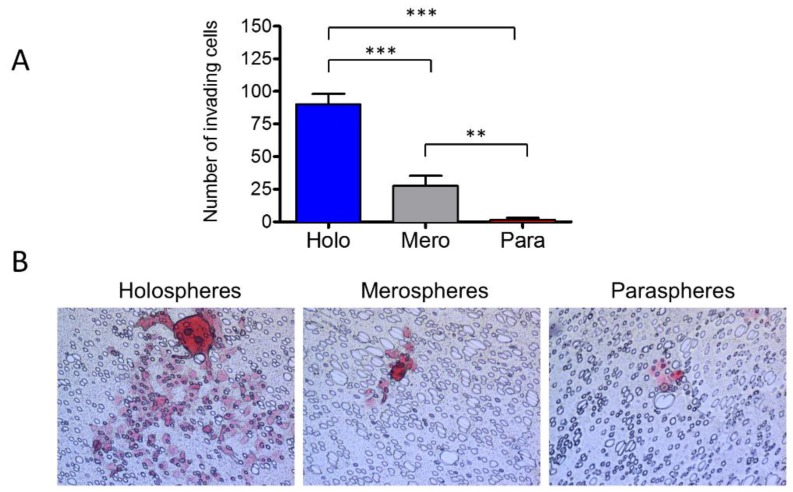
*Holospheres have increased invasive potential.* (**A** and **B**) Invasion assay of tumor spheres derived from holospheres, merospheres, and paraspheres. Holospheres show increased invasion compared to merospheres (*** *p* < 0.001) and paraspheres (*** *p* < 0.001). Merospheres invade significantly more than paraspheres (** *p* < 0.01).

We have shown that tumorspheres have different clonogenic potentials depending on their morphology; however, it is unknown whether aggressiveness of tumors differs among holospheres, merospheres and paraspheres. To better understand their role in cancer biology, we placed each sphere subtype into invasion chambers coated with fibronectin. The total number of spheres was adjusted to accommodate the same number of tumor cells per well with ratios of one holosphere for five merospheres and 12 paraspheres. After 8 h, holosphere-derived tumor cells were very efficient at invading the substrate compared to merosphere- and parasphere-derived tumor cells (Figure 5A,B) (*** *p* < 0.001). Merospheres produced a significant number of invading cells compared to paraspheres (** *p* < 0.01).

**Figure 6:**
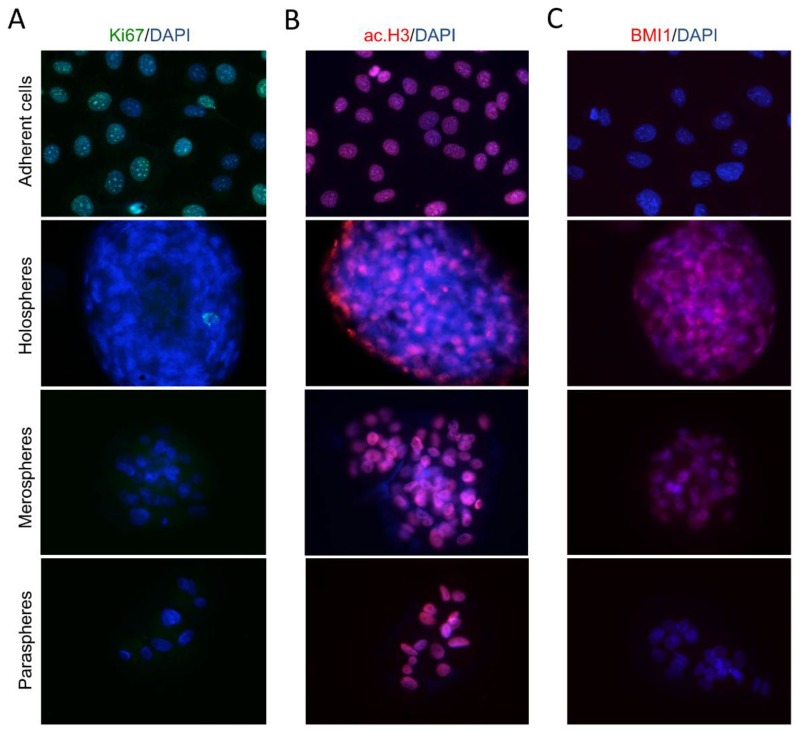
*Tumor spheres present reduced proliferation and express stem cell hallmarks.* (**A**) Proliferation of tumor spheres is reduced compared to tumor cells growing in adherent conditions (magnification × 200); (**B**) Histone acetylation of tumor spheres. Holospheres have reduced histone acetylation compared to merospheres, paraspheres and tumor cells growing in adherent conditions (magnification × 200); (**C**) Unlike paraclones and tumor cells growing in adhesion, holospheres and merospheres express BMI-1 (magnification × 200).

Culturing tumorspheres in ultra-low attachment substrates resulted in dramatic changes in CSC content, as we have previously demonstrated by FACS analysis (Figure 1A,E,F,G). Tumor cells growing in ultra-low adhesion conditions (spheres) have a lower Ki-67 proliferation rate compared to tumor cells growing as a monolayer (Figure 6A). In addition to cellular proliferation, tumorspheres are characterized by dramatic changes in chromatin organization (Figure 6B). Tumor cells grown in a monolayer have high levels of histone acetylation (ac.H3) that result in chromatin decondensation, increased transcription, and downregulation of DNA methylation. Holospheres have low levels of ac.H3 compared to merospheres, paraspheres and HNSCC cells grown in a monolayer. Histone deacetylation influences transcriptional gene silencing that is often observed in quiescent stem cells [27,28,29]. We also examined tumorspheres for expression of BMI-1, a transcriptional repressor that is upregulated in stem cells and associated with reduced survival of several types of tumors [30,31]. Interestingly, BMI-1 expression levels were high in holospheres and relatively high in the majority of tumor cells from the merospheres (Figure 6C). Paraspheres and tumor cells grown in a monolayer did not express BMI-1.

These findings suggest that holospheres contain a population of CSCs with greater “stemness” potential that will be of interest as a model for developing novel therapies targeting CSC.

## 3. Discussion

A better understanding of the biology and behavior of HNSCC will help guide the development of new therapeutic strategies. There are many difficulties inherent in managing head and neck cancer, and the lack of new therapies available for patients experiencing chemoresistance continues to directly impact the overall survival of patients. Many mechanisms underlie tumors acquiring resistance to therapeutics, including the acquisition of new mutations [32,33], activation of signaling pathways that increase resistance [34,35,36,37], and the presence of CSCs [38,39,40,41]. CSCs share many properties with normal stem cells. Like stem cells, CSCs are endowed with long-term self-renewal characteristics that are associated with a slow cell cycle [42,43]. These characteristics provide advantages over non-CSC tumor cells that are sensitive to conventional intercalating therapies that require cell division for maximum efficacy. Low cycling tumor cells are evident in holospheres, merospheres, and paraspheres, as we have demonstrated by Ki67 staining. In addition to mutation-driven cancer stem cells, epigenetic modifications play a dynamic role in tumor morphology, aggression, and invasion [44]. Epigenetic alterations characterized by histone modifications control gene transcription and cellular differentiation. Increased acetylation of histones results in chromatin decondensation, activation of transcriptional factors associated with cellular differentiation, and downregulation of DNA methylation. We have previously shown that HNSCC spheres cultured in ultra-low adhesion conditions do not maintain their spheroid structure upon pharmacological induction of histone acetylation using histone deacetylase inhibitors [44]. Interestingly, following continuous acetylation of histones, tumor cells grown in a monolayer undergo epithelial-mesenchyme transition (EMT) [44]. In contrast to histone acetylation, histone deacetylation induces chromatin condensation and transcriptional gene silencing that is often observed in quiescent stem cells [27,28,29]. Therefore, increased chromatin condensation likely plays a fundamental role in tumor resistance to chemotherapy. In fact, we can observe that the chromatin of tumor cells forming holospheres are in fact smaller than their counterpart merospheres and paraspheres, monolayer growing tumor cells (Figure 5C–E). The reduced nuclear size of holospheres corresponds to reduced levels of acetyl histone 3, as identified by immunofluorescence (Figure 6B). Although largely unknown, many mechanisms are able to induce tumor acetylation or deacetylation. For example, we have shown that chromatin in some HNSCC cell lines is acetylated in response to conditioned media from endothelial cells. In contrast, other HNSCC cells experience compact chromatin and reduced histone 3 acetylation in response to conditioned media from endothelial cells [44]. Given such distinct phenotypes, one could expect that dynamic chromatin conformation (acetylation status) in tumor cells may dictate resistance to therapy. Indeed, further analysis of a larger set of HNSCC cell lines showed that the intercalating agent cisplatin induces acetylation or deacetylation, depending on the cell line [37]. These findings suggest that not only are tumor cells epigenetically modified by environmental cues, such as conditioned media from endothelial cells, but they are also influenced by chemotherapeutic agents. The significance of this finding was revealed when tumor cells that showed chromatin deacetylation in response to cisplatin also had increased cisplatin resistance [37]. In contrast, tumor cells with acetylated chromatin in response to cisplatin were also sensitized to cisplatin. Therefore, deacetylation of histones that results in the transcription of stem cell-like genes and reduced cell cycle is associated with increased resistance to chemotherapy.

The activation of stem cell-like programs during histone deacetylation is also associated with the expression of BMI-1, a member of the polycomb repressor complex 1 that is involved in chromatin remodeling and is highly expressed in cancer cells and cancer stem cells [45,46,47]. Our data showed that holospheres, and to a certain extent merospheres, expressed BMI-1, unlike paraspheres and tumor cells grown in a monolayer; this suggests that holospheres are more undifferentiated, stem cell-like tumor cells (Figure 6C). Although more undifferentiated, and with tumor cells containing increased ALDH and CD44 expression, holospheres had superior ability to adhere to new substrates (Figure 2B,C) and to initiate cellular invasion (Figure 5B).

## 4. Materials and Methods

### 4.1. Cell Lineage

The Cal7, HN6, HN12, HN13 HNSCC cell line [48] were obtained from Dr. Silvio Gutkind. Tumor cell lines UMSCC17B, and UMSCC74A were obtained from Dr. Thomas Carey. All cells were cultured in Dulbecco's Modified Eagle's Medium (DMEM) supplemented with 10% fetal bovine serum, 100 units/mL penicillin, 100 μg/mL streptomycin, and 250 ng/mL amphotericin B. Cells were maintained in a 5% CO_2_-humidified incubator at 37 °C.

### 4.2. Tumor Sphere Formation Assay

HNSCC cells were plated on ultra-low attachment 6-well plates (Corning, New York, NY, USA) and grown for five days. Spheres were cultured in DMEM supplemented with 10% fetal bovine serum, 100 units/mL penicillin, 100 μg/mL streptomycin, and 250 ng/mL amphotericin B. Cells were maintained in a 5% CO_2_-humidified incubator at 37 °C. Sphere formation was observed daily with minimal disturbance. To classify and quantify by subtype, spheres grown in suspension were collected at day 5, transferred to a glass slide by centrifugation at 1500 rpm for 10 min at 4 °C using a cytospin system, and fixed with PFA for 15 min at room temperature (RT). Spheres were stained with hematoxylin and eosin and mounted in aqueous mounting media (Sigma, St. Louis, MO, USA). Under a microscope, clonal spheres were classified as holospheres, merospheres, or paraspheres based on size and borderline, as previously reported [10].

### 4.3. Immunofluorescence

Cells were placed on glass coverslips in 12-well plates and fixed with absolute methanol at −20 °C for 5 min. Spheres were prepared as described above. Cells and spheres were blocked with 0.5% (*v*/*v*) Triton X-100 in PBS and 3% (*w*/*v*) bovine serum albumin (BSA) and incubated with anti-Acetyl-Histone H3 (Lys9) (Cell Signaling, Danvers, MA, USA), anti-BMI-1 (Millipore, Billerica, MA, USA), and anti-Ki-67 (Cell Signaling, Danvers, MA, USA) as indicated. Cells were then washed three times and incubated with Fluorescein isothiocyanate (FITC) or Tetramethylrhodamine (TRITC)-conjugated secondary antibody for 60 min at RT and stained with Hoechst 33342 for visualization of DNA content. Images were captured using a QImaging ExiAqua monochrome digital camera attached to a Nikon Eclipse 80i Microscope (Nikon, Melville, NY, USA) and visualized with QCapturePro software.

### 4.4. Sphere–Driven Cell Invasion Assay

Holospheres, merospheres, and paraspheres from HN13 cells were isolated by pipetting spheres under a phase contrast microscope. They were seeded over a homogeneous thin layer of fibronectin (BD Biosciences, Bedford, MA, USA) in Millicell Cell Culture Inserts (Millipore, Billerica, MA, USA) containing a polycarbonate filter membrane with 8 μm-diameter pores in 24-well plates. The number of spheres seeded in each invasion chamber reflected a total of 1 × 10^3^ cells; therefore, the total number of spheres varied among holospheres, merospheres, and paraspheres. We previously determined the optimal invasion time of HNSCC tumor cells is 24 h from the time cells are seeded in the upper chamber to the presence of a substantial number of cells in the lower portion of the polycarbonate filter membrane (~60%–70% of the total area) [44]. DMEM supplemented with 20% FBS and 1% antibiotics was placed in the lower chamber. Cells were incubated for 24 h at 37 °C in a 5% CO_2_-humidified incubator. Invasive cells reaching the bottom of the filter membrane were stained with hematoxylin and eosin. Images were captured using a QImaging ExiAqua monochrome digital camera attached to a Nikon Eclipse 80i Microscope (Nikon, Melville, NY, USA) and visualized using QCapturePro software.

### 4.5. Flow Cytometry

CSCs derived from holospheres, merospheres, paraspheres, and HN13 cells were examined for ALDH activity using the Aldefluor kit (StemCell Technologies, Durham, NC, USA), according to the manufacturer’s instructions. During ALDH titration and routine FACS experiments, we used DEAB as the inhibitor of ALDH enzymatic activity to properly establish the DEAB negative cellular population. Single controls for ALDH and CD44 were also used to identify an independent population of tumor cells before the identification of double positive cells (ALDH+/CD44+). All samples were analyzed in a FACS Canto IV (BD Biosciences, Mountain View, CA, USA) at the University of Michigan Flow Cytometry Core.

### 4.6. Colony Formation Assay

Ten holospheres, merospheres, and paraspheres were individually isolated from low attachment culture conditions and seeded by sphere subtype into normal culture dishes and left to attach. The total number of colonies derived from each attached sphere was quantified.

### 4.7. CSC Clonogenic Potential Assay

A single cell suspension (2.5 × 10^3^) derived from dissociated holospheres, merospheres, or paraspheres was seeded in ultra-low adhesion conditions and cultured for 5 days. Spheres were centrifuged onto glass slides and quantified by subtype.

### 4.8. Long-Term Maintenance of Clonogenic Potential Assay

Ten holospheres, merospheres, and paraspheres were seeded under adherent culture conditions and maintained under monolayer growth for ten passages. Sample cells from passage number 2 and 10 were isolated and seeded in ultra-low adhesion conditions for 5 days. The number of holospheres, merospheres, and paraspheres were accessed at the end of the experiment.

### 4.9. Statistical Analysis

All statistical analysis was performed using GraphPad Prism (GraphPad Software, San Diego, CA, USA). Statistical analysis of total number of spheres derived from HN13 cells was performed using one-way ANOVA followed by Tukey’s Multiple Comparison Test. Tumor sphere attachment assay was performed using Two-way ANOVA following by Bonferroni posttest. The sphere adhesion assay was assessed by two-way analysis of variance (ANOVA) followed by the Bonferroni posttest. One-way ANOVA followed by Tukey’s multiple comparison tests was used to assess CSC clonogenic potential and the number of invading cells. Asterisks denote statistical significance (* *p* < 0.05; ** *p* < 0.01; *** *p* < 0.001; and ns *p* > 0.05).

## 5. Conclusions

Collectively, we have shown that HNSCC spheres growing in ultra-low adhesion conditions efficiently enrich the population of CSCs. However, not all CSCs are endowed with similar characteristics; for example, in contrast to merospheres and paraspheres, CSCs from holospheres generate all three subtypes of spheres. Tumor cells from holospheres also have superior ability to attach and invade compared to other sphere-derived tumor cells. These differences suggest that CSCs have distinct stages of differentiation within a tumor. Some CSCs have greater potential to generate progeny that self-renew and reconstitute all subtypes of spheres. Other CSCs only generate partially differentiated progeny with limited self-renewal and limited ability to reconstitute all subtypes of spheres. Nonetheless, tumor cells cultured in ultra-low attachment conditions are an exciting technique for amplifying CSCs and for studying the mechanisms underlying the acquisition of therapeutic resistance.
